# Clustering of Health Risk Behaviors in Mexican and Puerto Rican Men: Results from the Latino Men’s Health Initiative

**DOI:** 10.3390/nu14214495

**Published:** 2022-10-26

**Authors:** Angelica Alonso, Carlos E. Rosas, Alfred Rademaker, Lisa Sanchez-Johnsen

**Affiliations:** 1 School of Public Health, College of Medicine, University of Illinois at Chicago, Chicago, IL 60612, USA; 2 Department of Family and Preventive Medicine, Rush University Medical Center, Chicago, IL 60612, USA; 3 Department of Psychology, University of Illinois at Chicago, Chicago, IL 60607, USA; 4 Department of Preventive Medicine (Biostatistics), Feinberg School of Medicine, Northwestern University, Chicago, IL 60611, USA

**Keywords:** Hispanic, Latino, Latinx, health risk, diet, physical activity, smoking, men

## Abstract

Engaging in multiple health risk behaviors simultaneously may increase the risk for cardiometabolic diseases. This study examined the prevalence and clustering of three health behaviors (physical activity, fruit and vegetable consumption, and smoking) among Latino men. The participants were 99 Mexican and 104 Puerto Rican men who participated in a study addressing culture- and obesity-related factors. The health behaviors were obtained from self-reported and anthropometric assessments through objective measurements. Among all participants, 5% had no health risk behaviors, 30% had one, 47% had two, and 18% had all three; their most common health risk behavior cluster was low physical activity and low fruit and vegetable consumption (28%). Among Puerto Rican men, 7% had no health risk behaviors, 24% had one, 51% had two, and 18% had all three; their most common health risk behavior cluster was current smoker and low fruit and vegetable consumption (28%). Among Mexican men, 3% had no health risk behaviors, 36% had one, 43% had two and 19% had all three; their most common health risk behavior cluster was low physical activity and low fruit and vegetable consumption (33%). The findings highlight the need for lifestyle interventions that target multiple health risk behaviors related to cardiometabolic diseases in Latinos.

## 1. Introduction

Latinxs experience a disproportionate burden of cardiometabolic diseases, including high rates of hypercholesterolemia, obesity, diabetes, and hypertension [1,2]. The risk for and development of cardiometabolic diseases such as cardiovascular disease and diabetes are strongly influenced by modifiable health behaviors, particularly smoking, unhealthy diet, and low physical activity [3,4]. Although more attention has been paid to single health behaviors and their association with health [5], individuals often engage in more than one unhealthy behavior simultaneously (e.g., smoking and physical inactivity) [5], which may further increase their risk for chronic diseases. Indeed, prior studies have shown that the risk of chronic diseases and mortality increases with a greater number of health risk behaviors (see Lacombe et al. [6] for a review).

Moreover, extant research suggests that engagement in different health behaviors varies by race or ethnicity and sex. Specifically, men from racial or ethnic minorities generally have less favorable health behavior profiles than women and non-Hispanic or non-Latino White men [1,7]. For example, in general, men tend to engage in risky health behaviors more frequently and have higher odds of engaging in multiple behaviors simultaneously than women [8,9,10]. However, despite being part of the largest ethnic minority group in the United States (U.S.) [11], Latino men have seldom been included in studies focusing on clusters of health behaviors. Examining how health behaviors cluster together among this group may be an important step in developing culturally tailored, preventive interventions to improve cardiometabolic health, as changing simultaneously versus isolated occurring behaviors may require different strategies [12].

### 1.1. Health Behavior Clustering

Albeit not specifically among Latinxs, several studies have explored simultaneously occurring clusters of behaviors, primarily focused on smoking, drinking, poor diet, and low physical activity, which comprise the main behavioral determinants of chronic diseases [13]. Past studies have uncovered various clusters among these behaviors. For instance, using data from the U.S. National Health and Nutrition Examination Survey (NHANES) III, Berrigan et al. [7] identified 32 patterns of health behavior among a representative sample of non-Hispanic Whites, non-Hispanic Blacks, and Mexican Americans. The most common pattern was adhering to recommendations on alcohol and tobacco consumption while not adhering to recommendations for physical activity, dietary fat intake, or fruit and vegetable consumption [7]. While this was also the most common pattern among Mexican Americans, it was less prevalent (13.8%) than among non-Hispanic Whites (14.7%) and non- Hispanic Blacks (18.4%). A second pattern that was particularly more prevalent among Mexican Americans than other groups was non-adherence to physical activity and to fruit consumption recommendations and adherence to tobacco, alcohol, and vegetable recommendations. Berrigan et al. [7] also found that men, regardless of their race or ethnicity, were 2.6 times more likely to not adhere to all five recommendations (physical activity, fruit and vegetable consumption, alcohol, tobacco use, and dietary fat intake) than women.

Using data from the Aerobics Center Longitudinal Study (ACLS), Héroux et al. [14] identified two clusters of unhealthy behaviors among a primarily non-Hispanic White (95%) sample of 13,621 participants in the U.S. The first group was composed of individuals more likely to engage in smoking, alcohol use, unhealthy diet, and low physical activity, while the second one was more likely not to engage in any of the four unhealthy behaviors [14]. They also found that all behaviors were significantly associated with each other, such that engaging in one behavior was related to increased odds of engaging in another one. For instance, individuals with an unhealthy diet (relative to those with a healthy diet) were 2.45 times more likely to engage in low physical activity, 2.02 times more likely to smoke, and 1.61 times more likely to drink heavily [14].

Research studies from other countries further support for the clustering of health behaviors and the importance of considering sociodemographic differences. In a population-based study among Irish adults, for example, Conry et al. [15] found six different clusters of behaviors. Individuals in the healthy lifestyle cluster, characterized by non-smokers, high physical activity, healthy eating, and moderate alcohol use, tended to be women, older (65+ years), and of higher socioeconomic status (SES), while those in the mixed lifestyle cluster (non-smokers, moderate physical activity, and variable alcohol consumption) were more likely to be men, younger, and of low SES [15]. Similarly, using population-based data from 4238 German participants, Rabel et al. [16] identified three clusters of behaviors which varied by sex. The healthiest cluster (low to moderate drinking, favorable diet, moderate physical activity, and no smoking) was endorsed by women only, while the other two more heterogenous clusters were endorsed primarily by men (≥71%). Collectively, these studies suggest the co-occurrence of health behaviors is common, but patterns vary by sociodemographic factors. Men, in particular, seem to be more likely to engage in multiple risky behaviors than women; however, few studies have focused exclusively on men, and fewer on Latino men [17,18].

### 1.2. Health Behaviors in Latinxs

Past research on health behaviors among Latinxs has yielded mixed results. For instance, while some studies using self-reported data have found that fewer Hispanics/Latinos engage in less physical activity than non-Hispanic Whites [19,20], studies using objectively measured data indicate that Hispanics/Latinos engage in higher physical activity levels than non-Hispanic Blacks and Whites [21,22]. Similarly, some studies indicate that Hispanics have better-quality diets than non-Hispanic Blacks and Whites [23,24], while others find the opposite [19,25]. Moreover, although previous studies have found that smoking is not as prevalent among Hispanics/Latinos as it is among other racial/ethnic groups [7,26,27], these studies have focused primarily on Hispanics/Latinos of Mexican descent e.g., [7]. These differences may be explained by variations in diet, physical activity levels, and smoking rates between Latinx subgroups. Indeed, evidence from the Hispanic Community Health Study/Study of Latinos (HCHS/SOL) has revealed differences in diet quality [28], physical activity [29], and smoking [18] across Hispanic/Latino subgroups; thus, research on specific Latinx subgroups is warranted. In addition, it is imperative to examine the specific clustering of health behaviors, as certain combinations appear to be related to different health conditions. One study among ethnically diverse adults, for example, reported that not eating a healthy diet along with not doing vigorous physical activity was associated with an increased risk for hypertension, whereas not eating a healthy diet alone was a stronger predictor of diabetes [30].

Despite the high prevalence of health risk behaviors in Latinxs, especially men, very few studies have examined risk behavior clustering in this population. Moreover, clustering patterns may differ across ethnic and sex groups, and this may provide insight into the development of more effective health behavior interventions. To this end, the overall objective of this study was to examine the prevalence and clustering of three important health behaviors (smoking, fruit and vegetable intake, and physical activity) among Mexican and Puerto Rican men. We also examined whether these associations varied by Latino background.

## 2. Materials and Methods

### 2.1. Sample

This is a secondary analysis of data obtained from Mexican and Puerto Rican participants who enrolled in a cross-sectional study (R21CA143636) called the Latino Men’s Health Initiative (Iniciativa de Salud para Hombres Latinos). The Latino Men’s Health Initiative was an NIH-funded, community-based participatory research study [17]. The purpose of that cross-sectional parent study was to explore the role of four cultural variables (acculturation, acculturative stress, ethnic identity, and cultural values) that may help to explain the ethnic disparities in correlates of overweight or obesity (diet, physical activity, and body image) among Mexican and Puerto Rican men [17]. Eligibility criteria were as follows: Inclusion: (a) Mexican and Puerto Rican men. They could be biracial but had to identify as Mexican or Puerto Rican. (b) Between the ages of 18 and 65. (c) Those who agreed to provide informed consent. Exclusion: (a) Those with a lower body mass index (BMI) limit: <18.5 kg/m^2^. No upper BMI limit (as the target population of the larger study were men with normal weight, overweight, or obesity). (b) Those who were not able to comprehend English or Spanish. (c) Those who had an eating disorder, including bulimia nervosa, anorexia nervosa, and binge eating disorder. (d) Those who had plans to move from the Illinois area during the course of the study (i.e., 6 weeks).

The recruitment strategies, which are described in detail elsewhere [17], consisted of direct and indirect methods. The direct methods included in-person recruitment at Latinx organizations, churches, and community events. The indirect methods included newspaper and newsletter advertisements, website and listserv announcements, and letters to organizations with a Latino or health focus. All participants provided written informed consent. The data collected as part of the overall parent study and the analyses reported in the current study was approved by the Institutional Review Board (IRB) at the University of Illinois at Chicago (IRB-2011-0187). The parent study also received IRB approval from the research review board at Alivio Medical Center. In addition, the University of Illinois at Chicago served as the IRB of record for Northwestern University (STU00204427).

The participants completed anthropometric measures (height, weight, body fat, hip, waist), a health and culture interview, and a diet questionnaire and used an accelerometer for seven consecutive days. As noted elsewhere [17], 203 participants completed the measures and the health and culture interview, and 193 completed all study components.

### 2.2. Measures

#### 2.2.1. Demographics and Health-Related Variables

Sociodemographic, clinical, and health-related information was obtained from participants during a two and a half hour health and culture interview (see Sanchez-Johnsen et al. [17]). During the eligibility interview, the participants were asked, ‘What is your ethnic background?’ The response options included ‘Hispanic or Latino (can you specify which Hispanic or Latino ethnic group)’, along with ‘African American or Black (Not Hispanic)’; ‘White, Caucasian, or European (Not Hispanic)’; and five other categories. The participants were allowed to choose all that applied but had to specify that they were Mexican or Puerto Rican to continue participating in the study. In addition, the participants were asked to self-report their age, nativity, length of time lived in the U.S., highest grade completed, income, smoking status, and other socio-demographic background characteristics. For the purpose of this study, the length of time lived in the U.S. (≤20 years, 21–40 years, and 41–65 years), education (less than high school graduate, some high school education, high school graduate or equivalent/GED, some college education, and college graduate of a four-year university or graduate studies), and income (<$10,000, $10,000–$24,999, $25,000–$49,999 and ≥$50,000) were treated as ordinal variables. BMI was included as a categorical variable (categories defined below), and age was treated as a dichotomous variable with two categories (18–40 and 41–65 years).

#### 2.2.2. Body Mass Index

Weight status was assessed via BMI (weight (kg)/height (m)^2^) [31]. After removing their shoes, the participants’ height and weight were assessed using a SECA stadiometer (SECA, Chino, CA, USA) and SECA digital scale (SECA, Chino, CA, USA), which had been used in previous studies [32]. Weight status was defined as: normal weight: BMI = 18.5–24.99; overweight: BMI = 25–29.99; obese: BMI > 30 [31].

#### 2.2.3. Smoking Status

Smoking status was assessed using a single item from the Adult Stages of Change for Smoking questionnaire [33,34] which measured participants’ stage of change for quitting smoking. Participants were asked to respond to the question, ‘Do you currently smoke cigarettes?’ The answer was treated as a dichotomous variable to maintain uniformity within our analysis. The two categories were recoded and defined by ‘Yes’ (currently smoke) and ‘No’ (never smoked or quit smoking).

#### 2.2.4. Self-Reported Physical Activity

Physical activity was assessed via a single item from the Stages of Change for Exercise questionnaire [35,36], which measured participants’ stage of change for increasing exercise. Participants were asked to respond to the question, ‘Do you currently engage in regular physical activity?’ The following instructions were included: ‘For physical activity to be considered “regular” it must be done for 30 min at a time (or more) per day, and be done at least four days per week.’ Additional information about how physical activity was defined can be found in the studies by Hellsten et al. [35] and Nigg [36]. The response options were ‘Yes’ and ‘No’.

#### 2.2.5. Fruit and Vegetable Intake

Fruit and vegetable consumption was assessed using a single item from the Stages of Change for Fruit and Vegetable Intake survey, which measured the participants’ stage of change for increasing fruit and vegetable consumption [37,38,39]. The participants were asked to respond to the question, ‘How many servings of fruits and vegetables do you usually eat each day?’ The responses ranged from zero to six or more. In line with the established guidelines [40,41], the fruit and vegetable intake variable was dichotomized into ‘<5 servings/day’ or ‘≥5 servings/day’ for the purpose of this study.

### 2.3. Statistical Analyses

The descriptive statistics provided frequencies and percentages of participant characteristics and health risk behaviors, which were compared between Latino subgroups using the chi-square test. A sample size calculation was performed to achieve 80% power for overall correlation analyses and for comparison analyses between Latino descent groups.

Statistical analysis models were derived from the study by Baruth et al. [12]. The prevalence rates of (1) current smoking status, (2) physical activity, and (3) fruit and vegetable consumption were computed in the overall sample and in each Latino subgroup. Next, the prevalence of multiple health risk behaviors was calculated by assigning a score of 1 (versus 0) to each for being a smoker, engaging in less than 30 min of physical activity per day, and consuming <5 servings/day of fruits and vegetables. The number of risk factors for each person was the sum of these individual scores (score range 0–3).

A series of hierarchical log-linear models using SAS/STAT CATMOD 15.2 (SAS Institute, Cary, NC, USA) were fit to the observed counts using maximum likelihood estimation. First, the main effects model was used with the three risk behaviors. The model assumed mutual independence among the three risk behaviors. Then, one or more two-factor interactions were added to the model to obtain the model with the least significant likelihood ratio *p*-value (best fit as indicated by the least difference between the observed and expected frequencies). Within this best fit model, the most significant interaction term (i.e., the interaction term with the lowest *p*-value) identified the most important risk factor cluster. Any interaction included in the final model was interpreted as reflecting dependence among the risk behaviors. The models were calculated for all Latinos and for each Latino subgroup. For Puerto Rican models, 0.5 was used in place of the zero frequency where no Puerto Rican participants smoked and consumed at least 5 servings of fruits and vegetables.

## 3. Results

### 3.1. Demographic Characteristics

The demographic characteristics are described in detail elsewhere [17] and the key variables are summarized here. The sample included 203 Latino men (99 Mexicans and 104 Puerto Ricans) aged 18–65 years (*M* = 39.4; *SD* = 12.3). Most participants had lived in the U.S. more than 20 years (74%; *n* = 149), had acquired some college education (50%; *n* = 101), had income levels either less than $10,000 (29%; *n* = 59) or between $25,000–$49,000 (30%; *n* = 61), and had overweight or obesity as indicated by their BMI (66%; *n* = 135). The distribution of covariates remained relatively uniform across the Latino subgroups, except for age. Specifically, there were more Puerto Rican men (62%) aged 41–65 years than Mexican men (30%) of the same age group. The demographic information is reported in Table 1.

### 3.2. Prevalence of Health Risk Behaviors

The prevalence rates of each individual risk behavior, as well as the prevalence of the total number of risk behaviors, are shown in Table 2. Of the total sample of 203 Latino male participants, 38% smoked, 47% engaged in no physical activity, and 93% consumed <5 servings/day of fruits and vegetables. When examining the prevalence of multiple risk behaviors, 5% of the sample had no health risk behaviors, 30% had one, 47% had two, and 18% had all three health risk behaviors.

As previously reported by Sanchez et al. [17], 46% of the 104 Puerto Rican men smoked. In addition, the results from this study showed that among Puerto Rican men, 43% engaged in no physical activity, and 91% consumed <5 servings/day of fruit and vegetables. When examining the prevalence of multiple risk behaviors, 7% of the Puerto Rican sample had no health risk behaviors, 24% had one, 51% had two, and 18% had all three health risk behaviors.

As noted in Sanchez et al. [17], 29% of the 99 Mexican men smoked. In addition, the results from this study showed that among Mexican men, 52% engaged in no physical activity and 94% consumed <5 servings/day of fruit and vegetables. When examining the prevalence of multiple risk behaviors, 3% of the Mexican sample had no health risk behaviors, 36% had one, 43% had two, and 19% had all three health risk behaviors.

### 3.3. Clustering of Health Risk Behaviors

The prevalence rates of the eight possible health risk behavior clusters are shown in Table 3. The most common health risk behavior cluster was engaging in low physical activity and consuming <5 servings/day of fruits and vegetables (28%), followed by smoking and consuming <5 servings/day of fruits and vegetables (19%) and engaging in all three health risk behaviors (18%). Latino men did not have any frequencies for the categories of smoker with low physical activity and greater than or equal to 5 servings of fruits and vegetables.

The prevalence rates of six possible health risk behavior clusters for Puerto Rican men are shown in Table 4. The most common health risk behavior cluster was engaging in smoking cigarettes and consuming <5 servings/day of fruits and vegetables (28%), followed by low physical activity and consuming <5 servings/day of fruits and vegetables (23%), and lastly engaging in all three health risk behaviors (18%).

The prevalence rates of seven possible health risk behavior clusters for Mexican men are shown in Table 5. The most common health risk behavior cluster was engaging in low physical activity and consuming <5 servings/day of fruits and vegetables (33%), followed by engaging in all three health risk behaviors (17%) and lastly engaging in smoking and consuming <5 servings/day of fruits and vegetables (10%).

### 3.4. Modeling Results

The first log-linear model for Latino men assumed that the three risk behaviors were mutually independent of one another with a goodness of fit of χ^2^ (3) = 5.0, *p* = 0.17. The final (best fit) log-linear model containing smoking, fruit and vegetable intake, physical activity, the fruit and vegetable intake by physical activity interaction and the fruit and vegetable intake by smoking interaction had an improved goodness of fit of χ^2^ (1) = 0.11, *p* = 0.74. The most significant interaction term in the final model was the low physical activity by consuming <5 servings/day of fruits and vegetables interaction (*p* = 0.08), which corresponded to the most frequent cluster in the overall sample (28%). For Latino men in general, of the individuals who had less than 5 servings of fruit and vegetables per day (*n* = 188), 40% smoked and 49% did not engage in physical activity, while of the individuals who had 5 or more servings of fruit and vegetables per day (*n* = 15), 13% smoked and 20% did not engage in physical activity. Table 3 presents the observed frequencies and the expected frequencies under the independence and final models.

For Puerto Rican men, the mutually independent model had a goodness of fit of χ^2^ (4) = 10.1, *p* = 0.039. For the final (best fit) log-linear model containing smoking, fruit and vegetable intake, and physical activity, the fruit and vegetable intake and physical activity interaction and the fruit and vegetable intake and smoking interaction had an improved goodness of fit of χ^2^ (2) = 1.6, *p* = 0.45. The most significant interaction term in the final model was the smoking and consuming <5 servings/day of fruits and vegetables interaction (*p* = 0.04), which corresponded to the most frequent cluster in the Puerto Rican sample (28%). Of the individuals who had less than 5 servings of fruit and vegetables per day (*n* = 95), 45% did not engage in physical activity and 51% smoked, while of the individuals who had 5 or more servings of fruit and vegetables per day (*n* = 9), 22% did not engage in physical activity and 0% smoked. Table 4 presents the observed frequencies and the expected frequencies under the independence and final models.

For Mexican men, the mutually independent model had a goodness of fit of χ^2^ (3) = 3.6, *p* = 0.21. For the final (best fit) log-linear model containing smoking, fruit and vegetable intake, and physical activity, the fruit and vegetable intake and physical activity interaction had an improved goodness of fit of χ^2^ (2) = 1.6, *p* = 0.46. The most significant term in the final model was the low physical activity and consuming <5 servings/day of fruits and vegetables interaction (*p* = 0.21), which corresponded to the most frequent cluster in the Mexican sample (33%). For Mexican men, of the individuals who had less than 5 servings of fruit and vegetables per day (*n* = 93), 54% did not engage in physical activity, while of the individuals who had 5 or more servings of fruit and vegetables per day (*n* = 6), 17% did not engage in physical activity. Table 5 presents the observed frequencies and the expected frequencies under the independence and final models.

## 4. Discussion

We examined the prevalence and clustering of three common health behaviors among Mexican and Puerto Rican men. Our results revealed a high prevalence of smoking (38%), engaging in low levels of physical activity (47%), and particularly low consumption of fruits and vegetables (93%) among all participants. Among all participants, almost half (47%) engaged in two health risk behaviors while 30% engaged in one health risk behavior and 18% in all three health risk behaviors; only 5% did not engage in any health risk behavior. We also found differences based on Latino background. Specifically, fewer Puerto Rican (43%) relative to Mexican (52%) men engaged in low physical activity, although this difference was not significant. Lastly, more Puerto Ricans (51%) reported engaging in two health risk behaviors than Mexicans (43%); this difference, however, was not statistically significant.

These results align with the findings of previous studies. For example, the results of the HCHS/SOL revealed that the smoking rates were the highest (35.0%) among men of Puerto Rican descent compared to men of other Hispanic/Latino backgrounds [18]. Similarly, data from the Centers for Disease Control and Prevention demonstrated that between the periods of 2002–2005 and 2010–2013, the prevalence rates of smoking were the highest among Puerto Rican men (33.9%) compared to men of other Hispanic/Latino subgroups (22.5–27.6%), non-Hispanic White men (28.1%), and non-Hispanic Black men (31.7%) [42]. In addition, data from the HCHS/SOL showed that almost 50% of the men in the study engaged in less than 150 min per week of moderate to vigorous physical activity, with men of Puerto Rican descent having more minutes per day of moderate to vigorous physical activity, as assessed by an accelerometer, than men of other Hispanic/Latino backgrounds [29]. The previous studies have shown that Hispanics/Latinos do not meet the national recommendation for the consumption of fruits and vegetables [40,43,44], and the results of the HCHS/SOL revealed that individuals of Puerto Rican descent tend to eat less fruit and vegetables than other Hispanics/Latinos [45]. Overall, our findings provide further evidence of the high prevalence of smoking, physical inactivity, and low fruit and vegetable consumption among Mexican and Puerto Rican men.

Few studies have examined the prevalence of multiple risk behaviors among Latinxs; however, the few that have done so indicate that Hispanics/Latinos tend to engage in at least two risk health behaviors simultaneously [1,7]. In our study, almost half of all participants—and half of all Puerto Ricans—engaged in two health risk behaviors. These findings are somewhat similar to those of Daviglus et al. [1], which found that about half of all Hispanic/Latino men had at least two cardiovascular disease risk factors. Although Berrigan et al. [7] assessed adherence to recommendations rather than health risk factors, they found that the most common pattern among all participants, including non-Hispanic Blacks, non-Hispanic Whites, and Mexican Americans, involved non-adherence to health behavior recommendations regarding physical activity, fat intake, or fruit and vegetable consumption.

We also identified specific clusters of health risk behaviors. For instance, Latino men, in general, who did not engage in “regular” physical activity (i.e., 30 min or more of physical activity per day for at least four days per week) were more likely to consume <5 fruits and vegetables per day. The most common health risk behavior cluster in Puerto Rican men was engaging in smoking cigarettes and consuming <5 servings/day of fruits and vegetables (28%), whereas the most common health risk behavior cluster among Mexican men was engaging in low physical activity and consuming <5 servings/day of fruits and vegetables (33%). Furthermore, in Puerto Rican men, smoking was associated with less fruit and vegetables intake per day. These risk clusters were also verified through multivariate modelling.

To our knowledge, no study to date has examined health behavior clustering exclusively among Latinxs. The only study that included Latinxs in their sample—albeit only Mexican Americans—produced similar findings to ours, with clusters of adherence to recommendations for alcohol and tobacco but non-adherence to recommendations for physical activity, dietary fat intake, and fruit and vegetable consumption [7]. In the HCHS/SOL, the most common patterns of any two cardiovascular disease risk factors were hypercholesterolemia and obesity followed by hypercholesterolemia and smoking among Hispanics/Latinos of a Puerto Rican background; and hypercholesterolemia and obesity followed by hypercholesterolemia and hypertension among Hispanics/Latinos of Mexican descent [1]. Those patterns are roughly consistent with the patterns found in the current study, wherein smoking was more prevalent among Puerto Ricans and physical inactivity among Mexicans. Daviglus et al. [1], however, did not examine the clustering of health behaviors.

The awareness of health risk behaviors among Latino men has implications for health and health promotion. The low levels of physical activity and high prevalence of smoking coupled with low intake of fruits and vegetables are major risk factors for morbidity and mortality [3,46]. Conversely, higher consumption of fruits and vegetables along with nuts and whole grains has been associated with lower cardiovascular disease-related morbidity and mortality in men [47]. Hence, our findings underscore the need for interventions that target multiple health risk behaviors simultaneously. Although interventions that target multiple co-existing health risk behaviors are needed and are believed to have the potential for a greater public health impact [48], they are scant among Latinxs. Their scarcity may be due to lack of research on behavior clusters in this population. Our findings may provide a starting point for such interventions. The differences in behavior clusters between Mexican and Puerto Rican men identified in this study, for instance, may aid in tailoring interventions to the specific needs of these groups, thereby increasing their effectiveness.

A few limitations must be borne in mind. First, in this secondary data analyses, measures for physical activity, smoking, and fruit and vegetable intake were cross-sectional and self-reported. Indeed, past studies on physical activity have yielded conflicting results when using self-reported versus accelerometer data (e.g., [20,22]). In addition, twenty four hour food recalls and food diaries are more effective methods to obtain accurate measurements of fruit and vegetable intake [49]. Second, our study was limited to Mexican and Puerto Rican men from the Chicagoland area, which limits the generalizability to other Latinx subgroups and Mexicans and Puerto Ricans in other cities. At least one study has found differences in health risk behaviors between Mexicans and Puerto Ricans and their counterparts in different cities across the U.S. [18]. Future studies should address these limitations by using objective methods across multiple time points.

These limitations notwithstanding, our study had a number of strengths. The most important strength of this study is its focus on a greatly underserved and understudied group, namely Latino men, and particularly Mexican and Puerto Rican men—the two largest Hispanic/Latino ethnic groups in the U.S. [11]. This is important not only because of the scant research on this group and its large population size, but also because of the high rates of engagement in health risk behaviors and high prevalence of overweight and obesity. A second strength is the focus of the current study on the differences between Latino subgroups. Few studies outside of the HCHS/SOL have examined differences between Latinx subgroups, despite the glaring variations in health behaviors and disparities in health outcomes.

## 5. Conclusions

This is the first study to examine health risk behavior clustering exclusively among Latino men. Our findings showed that a large number of both Mexican and Puerto Rican men engaged in multiple health risk behaviors simultaneously, particularly low physical activity and low consumption of fruits and vegetables. In addition, there were notable differences in the most common clusters between Mexican and Puerto Rican men. Specifically, low consumption of fruits and vegetables along with low physical activity was more common among Mexican men, while low consumption rates of fruits and vegetables and smoking were more prevalent among Puerto Rican men. These findings have implications for preventing cardiometabolic diseases, as engaging in multiple health behaviors has been shown to be associated with cardiovascular disease, cancer, and all-cause mortality [6]. Moreover, these findings are of paramount importance for the development of effective behavioral change interventions tailored to these two groups. Future research is warranted to examine other health risk behaviors and the influence of social and cultural variables in health risk behaviors.

## 6. Authors’ Note

Parts of this manuscript were presented at the 2017 National Hispanic Medical Association Conference, the 2017 University of Illinois at Chicago Psychiatry Research Extravaganza, the 2018 National Hispanic Medical Association Conference, and the 2018 Medical Organization for Latino Advancement’s Latino Health Symposium. The data analyses reported in this publication were part of the first author’s MPH project.

The words ‘Hispanic’, ‘Hispanic/Latino’, and ‘Latinx’ are used interchangeably, depending on the study being reviewed. ‘Latinx’ is used as a general inclusive term to refer to all persons of Hispanic/Latin American background. The term ‘Latino’ is used for participants in the current study, as that was the term used in recruitment and interviews in the parent study (the Latino Men’s Health Initiative).

## Figures and Tables

**Table 1:** Characteristics of Latino men.

	Overall	Puerto Rican	Mexican	
Variable	*n* (%)	*n* (%)	*n* (%)	*p*
Country of Birth	203	104 (51)	99 (49)	
U.S., except Puerto Rico	114 (56)	62 (60)	52 (53)	
Puerto Rico	42 (21)	42 (40)	0	
Mexico	47 (23)	0	47 (47)	
Age				<0.0001
18–40	108 (53)	39 (38)	69 (70)	
41–65	95 (47)	65 (62)	30 (30)	
Length of U.S. Residence ^a^				<0.0001
0–20 y	52 (26)	14 (14)	38 (39)	
21–40 y	96 (48)	44 (43)	52 (53)	
41–65 y	53 (26)	45 (44)	8 (8)	
Education ^b^				0.09
Less than HS grad	18 (9)	6 (6)	12 (12)	
Some HS	29 (14)	21 (21)	8 (8)	
HS/HS Equivalent/GED	53 (26)	26 (25)	27 (27)	
Some College	59 (29)	29 (28)	30 (30)	
College Grad 4+	42 (21)	20 (20)	22 (22)	
Income ^c^				0.66
<$10,000	59 (29)	32 (31)	27 (27)	
$10,000–$24,999	48 (24)	21 (21)	27 (27)	
$25,000–$49,999	61 (30)	31 (30)	30 (30)	
≥$50,000	34 (17)	19 (18)	15 (15)	
BMI Status ^d^				0.79
Normal weight ^e^	68 (34)	37 (36)	31 (31)	
Overweight ^f^	70 (34)	34 (33)	36 (36)	
Obese ^g^	65 (32)	33 (32)	32 (32)	

Note: ^a^ Length of U.S. residence: Two people did not respond and were not categorized. ^b^ Education: HS = high school; GED = general educational development/diploma. Education: Two participants chose the option ‘Other’ and were not categorized. ^c^ Income: One participant did not respond and was not categorized. ^d^ BMI = body mass index. ^e^ BMI: 18.5–24.99; ^f^ BMI: 25–29.99; ^g^ BMI ≥ 30.

**Table 2:** Prevalence of health risk behaviors among Latino men (*n* = 203).

	Overall	Puerto Rican	Mexican	
Health Risk Behavior	*n* (%)	*n* (%)	*n* (%)	*p*
Smoking				0.01
Yes	77 (38)	48 (46)	29 (29)	
No	126 (62)	56 (54)	70 (71)	
Low Physical Activity ^a^				0.24
Yes	96 (47)	45 (43)	51 (52)	
No	107 (53)	59 (57)	48 (48)	
Low Fruits and Vegetables Intake				0.48
Yes (<5 servings/day)	188 (93)	95 (91)	93 (94)	
No (≥5 servings/day)	15 (7)	9 (9)	6 (6)	
# of Risk Behaviors				0.20
0	10 (5)	7 (7)	3 (3)	
1	61 (30)	25 (24)	36 (36)	
2	96 (47)	53 (51)	43 (43)	
3	36 (18)	19 (18)	19 (19)	

Note: ^a^ Currently do not engage in regular physical activity. # = Number.

**Table 3:** Clustering of health risk behaviors among Latino men (*n* = 203).

# of Risk Behaviors	Smoking (Y/N)	Low Physical Activity (Y/N)	Low Fruits and Vegetables Intake (Y/N)	Observed Frequency *n* (%)	Expected Frequency under Mutual Independence	Expected Frequency under Final Best Fit Model
3	Y	Y	Y	36 (18)	35.3	37.1
2	N	Y	Y	57 (28)	55.3	55.9
2	Y	N	Y	39 (19)	38.0	37.9
1	N	N	Y	56 (28)	59.5	57.1
1	N	Y	N	3 (2)	5.4	3.0
1	Y	N	N	2 (1)	3.7	2.0
0	N	N	N	10 (5)	5.8	10.0
2	Y	Y	N	0 (0)	0	0

Note: Latino men did not have any frequencies for the categories of smoker with low physical activity and greater than or equal to 5 servings of fruits and vegetables. Among all Latino men, the majority had 2 health risk behaviors consisting of low physical activity and low intake of fruits and vegetables per day. The final model indicates that there is a dependence of fruit and vegetable consumption on physical activity (*p* = 0.08) and smoking (*p* = 0.13). # = Number. Y = Yes. N = No.

**Table 4:** Clustering of health behaviors among Puerto Rican men (*n* = 104).

# of Risk Behaviors	Smoking (Y/N)	Low Physical Activity (Y/N)	Low Fruits and Vegetables Intake (Y/N)	Observed Frequency *n* (%)	Expected Frequency under Mutual Independence	Expected Frequency under Final Best Fit Model
3	Y	Y	Y	19 (18)	19.2	21.7
2	N	Y	Y	24 (23)	22.0	21.3
2	Y	N	Y	29 (28)	25.1	26.3
1	N	N	Y	23 (22)	28.7	25.7
1	N	Y	N	2 (1.9)	2.3	2.2
0	N	N	N	7 (6.7)	3.0	6.8

Note: Among Puerto Rican men, the majority had 2 health risk behaviors consisting of smoking and low fruit and vegetable servings per day. The final model indicates that there is a dependence of fruit and vegetable consumption on both smoking (*p* = 0.04) and physical activity (*p* = 0.04). # = Number. Y = Yes. N = No.

**Table 5:** Clustering of health risk behaviors among Mexican men (*n* = 99).

# of Risk Behaviors	Smoking (Y/N)	Low Physical Activity (Y/N)	Low Fruits and Vegetables Intake (Y/N)	Observed Frequency *n* (%)	Expected Frequency under Mutual Independence	Expected Frequency under Final Best Fit Model
3	Y	Y	Y	17 (17)	17.81	14.56
2	N	Y	Y	33 (33)	33.00	33.85
2	Y	N	Y	10 (10)	10.18	13.41
1	N	N	Y	33 (33)	32.01	31.18
1	N	Y	N	1 (1)	1.81	2.60
1	Y	N	N	2 (2)	1.01	1.03
0	N	N	N	3 (3)	3.18	2.38

Note: Among Mexican men, the majority had two health risk behaviors consisting of low physical activity and low fruit and vegetable servings per day. The final model indicates that there is a dependence of fruit and vegetable consumption on physical activity (*p* = 0.21). # = Number. Y = Yes. N = No.

## Data Availability

The data that support the findings of this study are available from the corresponding author upon reasonable request.
